# The Schizophrenia-Related Protein Dysbindin-1A Is Degraded and Facilitates NF-Kappa B Activity in the Nucleus

**DOI:** 10.1371/journal.pone.0132639

**Published:** 2015-07-14

**Authors:** Cheng Fu, Dong Chen, Ruijie Chen, Qingsong Hu, Guanghui Wang

**Affiliations:** 1 Laboratory of Molecular Neuropathology, Key Laboratory of Brain Function and Diseases and School of Life Sciences, University of Science and Technology of China, Chinese Academy of Sciences. Hefei, Anhui, China; 2 Laboratory of Molecular Neuropathology, Jiangsu Key Laboratory of Translational Research and Therapy for Neuro-Psycho-Diseases and College of Pharmaceutical Sciences, Soochow University, Suzhou, Jiangsu, China; 3 Department of Clinical Pharmacy and Pharmacology, the Second Affiliated Hospital of Wenzhou Medical University, Wenzhou, Zhejiang, China; National University of Singapore, SINGAPORE

## Abstract

Dystrobrevin-binding protein 1 (*DTNBP1*), a gene encoding dysbindin-1, has been identified as a susceptibility gene for schizophrenia. Functioning with partners in synapses or the cytoplasm, this gene regulates neurite outgrowth and neurotransmitter release. Loss of dysbindin-1 affects schizophrenia pathology. Dysbindin-1 is also found in the nucleus, however, the characteristics of dysbindin in the nucleus are not fully understood. Here, we found that dysbindin-1A is degraded in the nucleus via the ubiquitin-proteasome system and that amino acids 2-41 at the N-terminus are required for this process. By interacting with p65, dysbindin-1A promotes the transcriptional activity of NF-kappa B in the nucleus and positively regulates MMP-9 expression. Taken together, the data obtained in this study demonstrate that dysbindin-1A protein levels are highly regulated in the nucleus and that dysbindin-1A regulates transcription factor NF-kappa B activity to promote the expression of MMP-9 and TNF-α.

## Introduction

Dystrobrevin-binding protein 1 (*DTNBP1*), a gene encoding dysbindin-1, has been identified as a susceptibility gene for schizophrenia. Genetic variation in *DTNBP1* affects cognitive function in patients with schizophrenia [1, 2], and in the healthy population [2]. In schizophrenia patients, dysbindin-1 expression is reduced in the dorsolateral prefrontal cortex and during hippocampal formation [3, 4]. *Sandy* mice, which are deficient in dysbindin-1 expression, exhibit schizophrenia-related behavior [5–7].

Dysbindin is involved in neurotransmitter release and signal transduction [8–11]. Accumulating evidence shows that dysbindin affects nervous system development. By interacting with WAVE2 and Abi-1, dysbindin can stabilize dendritic protrusions, thus promoting dendritic spine maturation [12, 13]. Dysbindin also plays a role in the regulation of neurite outgrowth and in the development of growth cone morphology [14] and is a component of the lysosome-related organelles complex-1 (BLOC-1), which regulates intracellular membrane trafficking and interacts with soluble *N*-ethylmaleimide-sensitive factor attachment protein receptor (SNARE) proteins to promote neurite outgrowth [15]. Dysbindin also interacts with necdin in the cytoplasm, which represses the role of p53 in the nucleus, to promote neurite outgrowth [16].

In addition to its functions in the cytoplasm, dysbindin acts in the nucleus [17–19]. As a nuclear export signal occurs in dysbindin, this molecule is apparently diffused in the cytoplasm. However, dysbindin is distributed in the nucleus following the administration of the nuclear export inhibitor leptomycin B [18]. Although some nuclear interaction partners have been identified, the exact role of dysbindin in the nucleus remains unknown. Interestingly, the expression of the synaptic vesicle phosphoprotein synapsin I is regulated by dysbindin in the nucleus [8, 19]. Synapsin impacts neurotransmitter release and is lower during hippocampal formation in patients with schizophrenia [20]. Therefore, dysregulation of dysbindin in the nucleus might contribute to the pathogenesis of schizophrenia.

NF-kappa B plays a key role in regulating inflammation-related molecules and has been linked to a wide range of diseases including nervous system disorders. In the nervous system, NF-kappa B is present in glial cells and neurons. In neurons, as a transcription factor, NF-kappa B is involved in cell survival, neuronal process growth and synaptic plasticity. Inhibition of NF-kappa B activity represses neurite outgrowth in peripheral and cortical neurons [21] and increases spinal and excitatory synapse density in hippocampal neurons during development or in response to appropriate stimuli [22]. Treatment with NF-kappa B inhibitor impairs long-term fear memory [23] and long-term spatial memory [24]. Furthermore, NF-kappa B knockout mice demonstrate selective memory deficits [25–27]. Thus, NF-kappa B signaling is involved in neuronal development and memory. Most recently, NF-kappa B activity has been reported to be significantly decreased in the brains of schizophrenia patients [28], suggesting that NF-kappa B is associated with schizophrenia.

Tripartite motif protein 32 (TRIM32) exhibits E3 activity and plays a role in the ubiquitination of dysbindin in the cytoplasm [29]. TRIM32 regulates dysbindin degradation in transfected cells, and both of these proteins colocalize with α-actinin. These data provide evidence that dysbindin might be degraded by the ubiquitin-proteasome system.

In this study, we identified that dysbindin protein levels are highly regulated in the nucleus and that the degradation of this protein is associated with amino acids 2–41 at the N-terminus. Dysbindin-1A interacts with NF-kappa B in nucleus; and the nuclear dysbindin-1A promotes the transcriptional activity of NF-kappa B. Thus, our data identify a novel function for dysbindin in the nucleus.

## Materials and Methods

### Recombinant DNA construction

The dysbindin-1A plasmids have been described previously [19]. To prepare the pEGFP-N3-NLS-dysbindin-NES mutant, the dysbindin^L243A, I246A, L252A, L256A^ fragment was excised from pEGFP-N3-dysbindin^L243A, I246A, L252A, L256A^ using *Bgl II/EcoR I* and inserted into pEGFP-N3-NLS, which was cleaved using the same enzymes. pEGFP-C2-dysbindin-1A 1-189 was constructed using PCR and the primers 5’-CGGGATCCCAATGCTGGAGACCCTTC-3’ and 5’-ACGCGTCGACTTATTGCTGGGTGTGCTC-3’; the PCR products were then inserted into pEGFP-C2 at *Bgl II/Sal I* sites. The NF-kappa B subunit p65 plasmid EGFP-p65 was kindly provided by Dr. Yi-Zheng Wang (Institute of Neuroscience, Chinese Academy of Sciences, Shanghai, China); this plasmid was subcloned into pET-15b using the primers 5’-CCGCTCGAGATGGACGAACTGTTCCCCCTC-3’ and 5’-CGGGATCCGGAGCTGATCTGACTCAG-3’ at *Xho I*/*BamH I* sites, or p3×flag-myc-cmv^24+^ with 5’-GGGGTACCAATGGTCGAACTGTTCCCC-3’ and 5’-GCTCTAGAGGAGCTGATCTGACTCAGCAG at *Kpn I/Xba I* sites. The dysbindin-1A 206–351 and 1–293 was amplified using primers 5’-GAAGATCTGCAATGGAGCAGTACCTGTCC-3’ and 5’-CCGGAATTCAAAGAGTCGCTGTCCTC-3’, and 5’-GAAGATCTGCAATGCTGGAGACCCTTC-3’ and 5’-CGACGTCGACGGCTCTTAATTCTGAG-3’, respectively. The PCR products were inserted into *Bgl II/EcoR I* or *Bgl II/Sal I* sites in pEGFP-N3-NLS plasmid.

The remaining mutant plasmids were created using a site-directed mutagenesis kit (Takara, Otsu, Shiga, Japan). pEGFP-N3-NLS-dysbindin-1A K21R was constructed using the primers 5’-AGTGACAAGTCAAGAGAAGCA-3’ and 5’-TAAAGTCCTCAGCCCGGAGGT-3’, and pGEX-5x-1-dysbindin^Δ2–41^ was constructed using the primers 5’-TTGCCAAAGTACTCTGCTGGA-3’ and 5’-CATTGGGATCCCACGACCTTC-3’. The pEGFP-C2-dysbindin^Δ2–41^ product was created by excising the dysbindin^Δ2–41^ cDNA from pGEX-5x-1-dysbindin^Δ2–41^ using *BamH I/Xho I* sites and was inserted into *Bgl II/Xho I*-cleaved pEGFP-C2. The pEGFP-C2-NLS-dysbindin^Δ2–41^ was created with PCR using primers 5’-AGAGGAAAGTGCAGATCCCAATGTTGCCA-3’ and 5’-TCTTTTTGGGAGTCCGGCCGGACTTGTA-3’. HA-Ub was a gift from Dr. Wu Mian (University of Science and Technology of China, China).

### Cell culture, transfection, and immunocytochemical analyses

HEK293 and Neuro2A (N2a) (Cell bank of Chinese Academy of Sciences, Shanghai) cells were cultured in Dulbecco’s modified Eagle’s medium (DMEM) (GIBCO, Grand Island, NY, USA) containing 10% fetal bovine serum (FBS) (GIBCO), 100 μg/ml penicillin and 100 μg/ml streptomycin. SH-SY5Y cells were grown in DMEM/F-12 (GIBCO) containing 10% FBS. The cultured cells were washed with 1×PBS buffer and then transfected with expression plasmids using Lipofectamine 2000 (Invitrogen, La Jolla, CA, USA) in DMEM according to the manufacturer’s recommendations. For immunocytochemical staining, cells transfected with the expression vectors were grown on cover slides and then fixed with 4% paraformaldehyde for 5 minutes at room temperature; then, the cells were incubated with 0.25% Triton X-100 for 5 minutes and blocked with 0.1% FBS in PBS. The nuclei were stained with DAPI (Sigma, Saint Louis, MO, USA). The samples were observed using an inverted system microscope (IX7; Olympus, Tokyo, Japan).

### Immunoprecipitation experiments

HEK293 cells expressing the indicated plasmids or SH-SY5Y cells were cultured for 36 hours and then treated or not treated 10 μM MG132 (Sigma, Saint Louis, MO, USA) for 12 hours before harvesting. The cells were sonicated in cell lysis buffer containing 50 mM Tris-HCl (pH 7.5), 150 mM NaCl, 1% NP-40, and 0.5% sodium deoxycholate, supplemented with protein inhibitor cocktail (Roche, Mannheim, Germany). Cellular debris was removed by centrifugation at 10,000 *g* for 30 minutes at 4°C. The supernatants were incubated with antibodies and protein G Agarose (Roche, Mannheim, Germany) overnight at 4°C. The beads were washed with cell lysis buffer, bound proteins were then eluted with SDS sample buffer and subjected to immunoblot analysis.

### GST-pulldown assay

GST, GST-dysbindin-1A and His-p65 were expressed in *E*. *coli*. GST and GST-dysbindin-1A were then incubated with Glutathione-Sepharose 4B (GE Healthcare, Pittsburgh, PA, USA) for 1 hour at 4°C and washed three times with 1×PBS. The washed Glutathione-Sepharose 4B was incubated with His-p65 for 3 hours at 4°C and then washed six times with 1×PBS to remove unbound proteins. Bound proteins were eluted with SDS sample buffer and subjected to immunoblot analysis.

### RNA interference analysis

The double-stranded oligonucleotides si-dys 1# 5’-AAGUGAUAAGUCAAGAGAAGCAAdTdT-3’ and si-dys 2# 5’- UGGCAAGCCUGGCUCAUUUdTdT-3’ were used against murine dysbindin-1A. The negative control used random double-stranded oligonucleotides. The primers were synthesized by Shanghai GenePharma (Shanghai, China) and transfected using Lipofectamine RNAi MAX Reagent (Invitrogen, Waltham, MA, USA) in Opti-MEM (GIBCO, Waltham, MA, USA) according to the manufacturer’s instructions. The cells were cultured for 48 hours and then analyzed.

### RNA isolation and real-time PCR

Total RNA was isolated using TRIzol (Invitrogen). Then, the RNA was reverse transcribed into cDNA using the TranScript First-Strand cDNA Synthesis Kit (Takara, Shiga, Japan). Real-time PCR was performed using SYBR Green PCR Master Mix (Applied Biosystems) and an Applied Biosystems 7500 Fast Real-time PCR System. The following primers were used to amplify the target genes: (1) PRKACA 5’-GGCTCTCGGAGTCCTCATC-3’ and 5’-CAGAGCTGAAGTGGGATGG-3’; (2) MMP-9 5’-GTCTTCCTGGGCAAGCAGTA-3’ and 5’-CTGGACAGAAACCCCACTTC-3’; and (3) TNF-α 5’-CATCTTCTCAAAATTCGAGTGACAA-3’ and 5’-TGGGAGTAGACAAGGTACAACCC-3’.

### Dual-luciferase reporter assay

Neuro2A cells were co-transfected with NF-kappa B reporter plasmids (pGL6-NFκB luc, Beyotime, Shanghai, China) encoding Renilla (Promega) using Lipofectamine 2000. After transfection, RNA oligonucleotides were added to culture medium containing 10% FBS. The results are presented as the means ± standard error (S.E.) of three independent transfection experiments.

### Immunoblot analysis

Proteins were subjected to SDS-PAGE and then transferred onto a polyvinylidene difluoride membrane (Millipore, Billerica, MA, USA). The following primary antibodies were used: (1) an anti-dysbindin antibody [19], (2) a monoclonal anti-GFP antibody (Santa Cruz Biotechnology, Santa Cruz, CA, USA), (3) an anti-Ub antibody (Santa Cruz Biotechnology), (4) an anti-HA antibody (Santa Cruz Biotechnology), (5) an anti-Flag antibody (Sigma), (6) an anti-p65 antibody (Abcam), (7) an anti-Histone H2B antibody (Abcam) and (8) a monoclonal anti-GAPDH antibody (Millipore). Anti-mouse IgG-HRP and anti-rabbit IgG-HRP antibodies (GE Healthcare, UK) were used as secondary antibodies. The proteins were visualized using a SuperSignal West Pico instrument (Thermo Scientific, Waltham, MA, USA).

### Degradation assay

Twenty-four hours after transfection, HEK293 cells expressing the indicated plasmids were divided into four groups. After a further 24 hours of culture, the cells were treated with 100 μg/ml cycloheximide (CHX) (Sigma, Saint Louis, MO, USA) to inhibit protein synthesis or were pre-treated with ethanol or 20 ng/ml leptomycin B (LMB) (Beyotime, Shanghai, China) for 1 hour before CHX was added. The cells were harvested after 0, 3, 6 and 9 hours of CHX treatment.

### Statistical analysis

Relative density analysis of immunoblots from three independent experiments was performed using Adobe Photoshop CS8.0 (Adobe, San Jose, CA, USA). The final data were analyzed using Origin 6.0 software (Originlab, Northampton, MA).

## Results

### Nuclear degradation of dysbindin-1A

**Fig 1:**
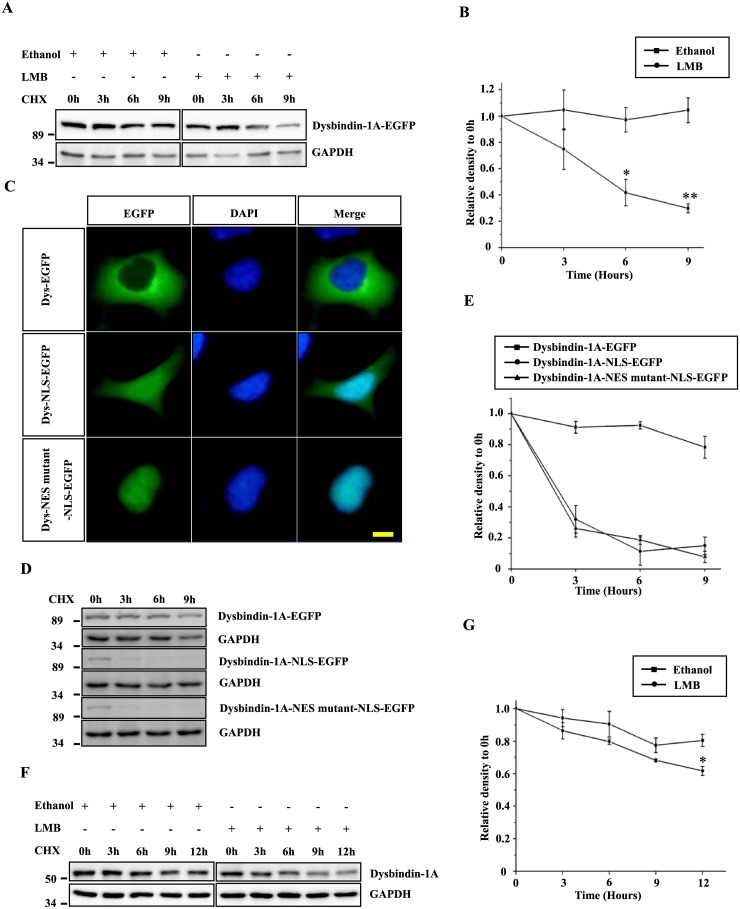
Dysbindin-1A is degraded in the nucleus. (A) HEK293 cells that had been transfected with dysbindin-1A-EGFP were pre-treated with leptomycin B 20 ng/ml or equal volumes of ethanol for 1 hour, respectively. The cells were then treated with CHX for the indicated time, and cell extracts were subjected to immunoblot analysis. (B) The band intensity of dysbindin-1A-EGFP relative to GAPDH is shown. The values shown represent the means ± S.E. of three independent experiments. *, *P*<0.05; **, *P*<0.01; one-way ANOVA. (C) Subcellular localization of dysbindin-1A-EGFP and its variants that harbor a nuclear localization signal and/or a nuclear export signal mutant. HEK293 cells were transfected with the indicated plasmids, and the nuclei were stained with DAPI; The bar represents 10 μm. (D) The half-life of dysbindin-1A-NLS or the NES mutant was shorter than that of wild type dysbindin-1A. Dysbindin-1A-EGFP, dysbindin-1A-NLS-EGFP and dysbindin-1A-NES mutant-NLS-EGFP were transfected into HEK293 cells for 24 hours; the cells were then treated with CHX (100 μg/ml) for the indicated time. (E) The data from three independent experiments of (D) were quantified. The values shown represent means ± S.E. (F) The HEK293 cells were pre-treated with leptomycin B 20 ng/ml or equal volumes of ethanol for 1 hour, respectively. The cells were then treated with CHX for the indicated time, and cell extracts were subjected to immunoblot analysis. (G) The quantified analysis from three independent experiments of (F). The values shown represent the means ± S.E. *, *P*<0.05; one-way ANOVA.

Because dysbindin-1A can localize to the nucleus, we determined whether this localization affects its degradation. HEK293 cells expressing dysbindin-1A-EGFP were pretreated with leptomycin B (LMB) to inhibit nuclear export. When protein synthesis was inhibited by cycloheximide (CHX), an increased degradation of dysbindin-1A-EGFP was observed in the LMB-treated cells (Fig 1A and 1B). Furthermore, dysbindin-1A-NLS-EGFP and dysbindin-1A-NES mutant-NLS-EGFP, two forms of dysbindin-1A that showed nuclear localization (Fig 1C), were degraded more rapidly than wild type dysbindin-1A (Fig 1D and 1E). The degradation of endogenous dysbindin-1A in HEK293 cells was also significantly enhanced after a treatment of LMB, a drug that inhibits the nuclear export protein exportin 1 to increase the nuclear dysbindin-1A (Fig 1F and 1G). These data suggest that dysbindin-1A is degraded more rapidly within the nucleus.

### Dysbindin-1A degradation via the ubiquitin-proteasome pathway in the nucleus

**Fig 2:**
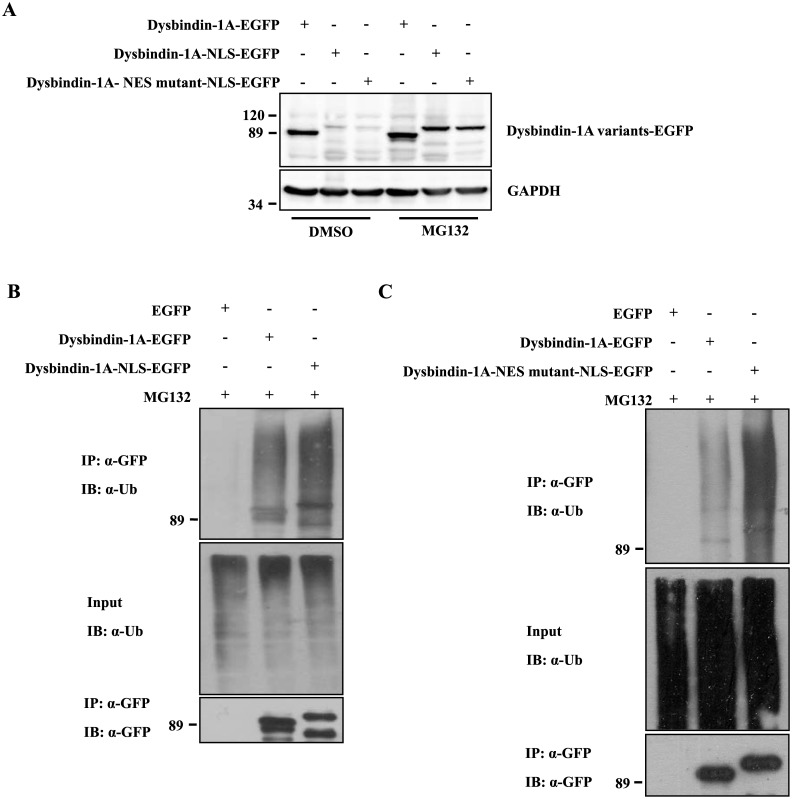
Dysbindin-1A is degraded via the ubiquitin-proteasome pathway in the nucleus. (A) Nuclear dysbindin-1A was degraded via the ubiquitin-proteasome pathway. Twenty-four hours after transfection with dysbindin-1A-EGFP, dysbindin-1A-NLS-EGFP and dysbindin-1A-NES mutant-NLS-EGFP, cells were treated with DMSO or MG132 (10 μM) for 12 hours, respectively. (B and C) Dysbindin-1A-NLS-EGFP and dysbindin-1A-NES mutant-NLS-EGFP were transfected into HEK293 cells for 24 hours. The cells were then treated with MG132 (10 μM) for 12 hours. Cell lysates were immunoprecipitated with GFP antibody and immunoblotted with ubiquitin antibody.

The 26S proteasome complex is present in the nucleus [29, 30], and we observed that dysbindin-1A degradation is increased in the nucleus. We wondered whether dysbindin-1A is degraded by the ubiquitin-proteasome system in the nucleus. HEK293 cells expressing dysbindin-1A-EGFP, dysbindin-1A-NLS-EGFP or dysbindin-1A-NES mutant-NLS-EGFP were treated with DMSO or MG132 for 12 hours and then subjected to immunoblot analysis against an anti-GFP antibody. As shown in Fig 2A, the levels of all three types of dysbindin-1A were increased after MG132 treatment. Moreover, the levels of dysbindin-1A-NLS-EGFP and dysbindin-1A-NES mutant-NLS-EGFP were markedly higher after MG132 treatment, suggesting that dysbindin-1A is subjected to proteasomal degradation in the nucleus. Because ubiquitination is essential for substrate degradation by the proteasome, we examined the ubiquitination of dysbindin-1A-EGFP, dysbindin-1A-NLS-EGFP, and dysbindin-1A-NES mutant-NLS-EGFP. HEK293 cells expressing the appropriate three plasmids were treated with MG132 for 12 hours. The proteins were then immunoprecipitated from the cell lysate supernatants using an anti-GFP antibody and tested against an anti-Ub antibody (Fig 2B and 2C). Dysbindin-1A-NLS-EGFP and dysbindin-1A-NES mutant-NLS-EGFP were ubiquitinated at higher levels than dysbindin-1A-EGFP. These data support the hypothesis that dysbindin-1A is degraded by the ubiquitin-proteasome pathway in the nucleus.

### The N-terminus of dysbindin-1A is important for its degradation

**Fig 3:**
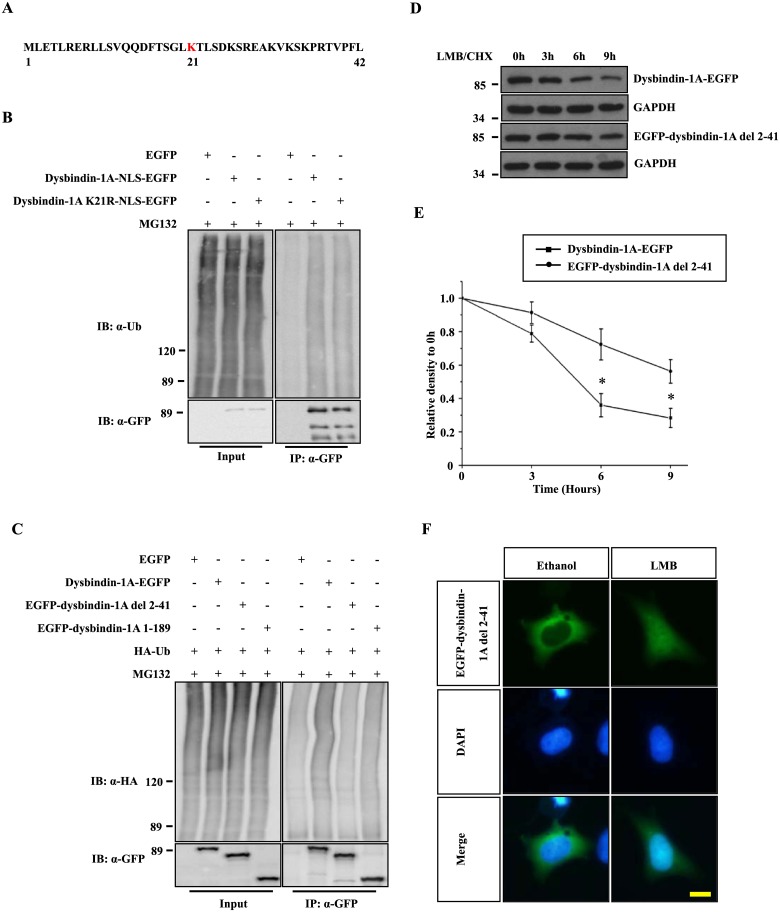
The N-terminal 2–41 amino acids of dysbindin-1A are important for its nuclear degradation. (A) The N-terminal sequence of dysbindin-1A (amino acids 1–42). The sequence was scored using Ubpred and BDM-PUB, and potential ubiquitination sites are colored red. (B) The potential dysbindin-1A ubiquitination site, lysine 21, was mutated to arginine. The ubiquitination of K21R and WT forms of dysbindin-1A was examined. (C) Dysbindin-1A-EGFP, EGFP-dysbindin (residues 1–189) or EGFP-dysbindin-1A residue 2–41 deletion mutant were co-transfected with HA-Ub into HEK293 cells, respectively. After culturing for 24 hours, 10 μM MG132 was added, and the cells were then cultured for an additional 12 hours. After lysis, the proteins were immunoprecipitated using an anti-GFP antibody and immunoblotted with an HA antibody. (D) The EGFP-dysbindin-1A residue 2–41 deletion mutant was more stable in the nucleus. HEK293 cells expressing dysbindin-1A-EGFP or EGFP-dysbindin-1A residue 2–41 deletion mutant were treated with leptomycin B (20 ng/ml) for 1 hour. The cells were then treated with CHX for the indicated time. Finally, cell extracts were subjected to immunoblot analysis. (E) The band intensity of the dysbindin-1A-EGFP and EGFP-dysbindin-1A residue 2–41 deletion mutant is shown, relative to GAPDH. The values shown represent the means ± S.E. of three independent experiments. *, *P*<0.05; one-way ANOVA. (F) The localization of EGFP-dysbindin-1A del 2–41 under treatment of Ethanol or LMB. The nuclei were stained with DAPI; The bar represents 10 μm.

Because dysbindin-1A is ubiquitinated (Fig 2), we predicted the potential dysbindin-1A ubiquitination site(s) using two ubiquitination prediction programs: UbPred and BDM-PUB [31, 32]. Lysines at the N-terminus were predicted as potential ubiquitination sites, and lysine 21 was predicted to be a ubiquitination site by both programs (Fig 3A). Thus, we created a dysbindin-1A K21R-NLS-EGFP construct in which lysine 21 was mutated to arginine. We transfected cells with dysbindin-1A-NLS-EGFP and dysbindin-1A K21R-NLS-EGFP plasmids and then treated the cells with MG132. However, we did not observe any differences in ubiquitination between the dysbindin-1A-NLS-EGFP and dysbindin-1A K21R-NLS-EGFP proteins (Fig 3B), suggesting that dysbindin-1A K21 might not be important for ubiquitination.

**Fig 4:**
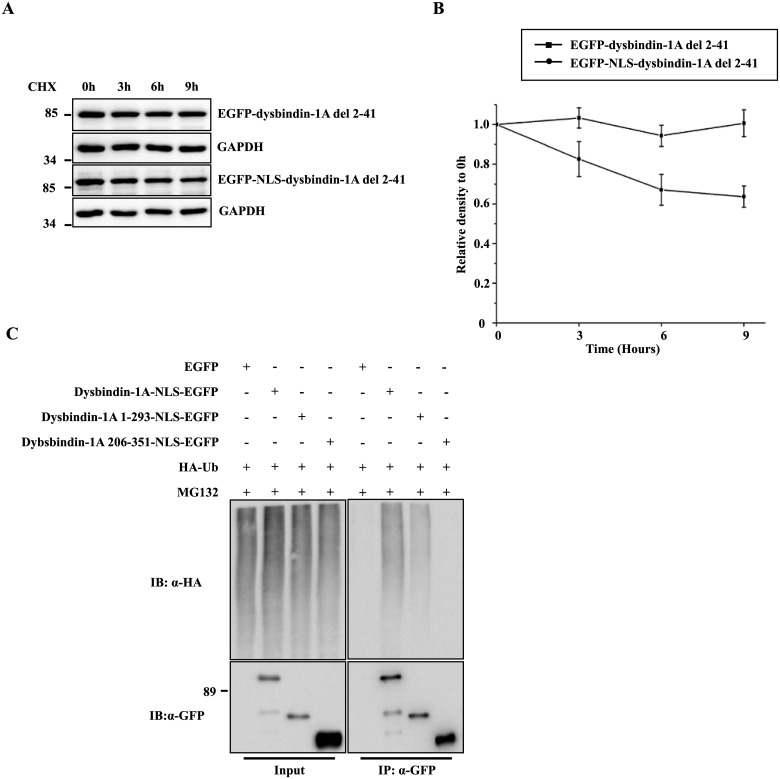
EGFP-NLS-dysbindin-1A del 2–41 is stable in nucleus. (A) The degradation of EGFP-dysbindin-1A del 2–41 or EGFP-NLS-dysbindin-1A del 2–41. (B) The quantitative analysis of (A). The values shown represent the means ± S.E. of three independent experiments. (C) The C-terminus of dysbindin-1A was not ubiquitinated in nucleus. The deletion mutants of dysbindin were transfected into cells. The ubiquitination of deletion mutants were examined.

We next created a dysbindin-1A construct in which amino acids 2–41 were deleted. When cells were transfected with the EGFP-dysbindin-1A del 2–41 plasmid, EGFP-dysbindin-1A del 2–41 was ubiquitinated at lower levels than either dysbindin-1A-EGFP or EGFP-dysbindin-1A 1–189 (Fig 3C). Furthermore, steady-state levels of EGFP-dysbindin-1A del 2–41 were higher than those of the full-length form after LMB treatment (Fig 3D and 3E), although a treatment of LMB still induced a nuclear translocation of EGFP-dysbindin-1A del 2–41 (Fig 3F). In addition, with a NLS, steady-state levels of EGFP-NLS-dysbindin-1A del 2–41 were also higher than those of the full-length form (dysbindin-1A-EGFP) in nucleus (Fig 4A and 4B). These data suggest that N-terminal amino acids 2–41 might be required for dysbindin-1A degradation in the nucleus. It was reported that there is a PEST domain in the C-terminus of dysbindin-1A, which is required for its proteasomal degradation [33]. We therefore created the deletion mutants with NLS to identify the ubiquitination. There was no ubiquitination of the C-terminus that contains the PEST domain, however, the ubiquitination of the N-terminus was decreased as compared to that of the full-length form (Fig 4C), suggesting that the C-terminus with the PEST domain may facilitates the ubiquitination of the N-terminus, although itself is not ubiquitinated.

### Dysbindin-1A interacts with NF-kappa B (p65) in nucleus and promotes its transcription activity

**Fig 5:**
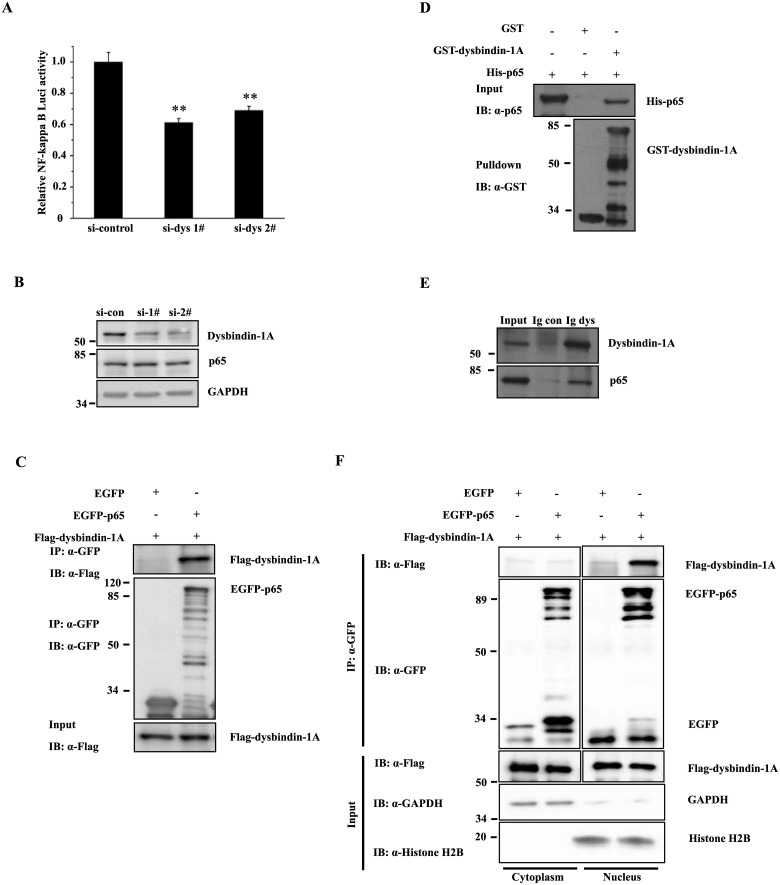
Dysbindin-1A interacts with Rel A (p65) and promotes NF-kappa B activity. (A) A dual luciferase reporter assay shows that dysbindin-1A promotes NF-kappa B transcriptional activity. (B) Dysbindin-1A knockdown does not influence the protein levels of p65 in N2a cells. N2a cells were transfected with si-dys1# or si-dys2#. The resulting cell lysates were subjected to immunoblot analysis using antibodies to dysbindin-1A or p65. (C) p65 interacts with Flag-dysbindin-1A. In HEK293 cells that were co-transfected with Flag-dysbindin-1A and EGFP-p65, EGFP or EGFP-p65 was immunoprecipitated using an anti-GFP antibody. The immunoprecipitants were subjected to immunoblot analysis with the indicated antibodies. (D) Dysbindin-1A interacts with p65 in a GST-pulldown assay. (E) Endogenous dysbindin-1A interacts with endogenous p65 in SH-SY5Y cells. (F) EGFP-p65 interacts with Flag-dysbindin-1A in nucleus. The cytoplasm fraction or nuclear fraction of HEK293 cells transfected with EGFP/EGFP-p65 and Flag-dysbindin-1A were immunoprecipitated using an anti-GFP antibody. The immunoprecipitants were subjected to immunoblot analysis with the indicated antibodies. *IB*, immunoblot; *IP*, immunoprecipitation.

The transcription factor NF-kappa B plays a role in many cellular functions. We wondered whether crosstalk exists between the dysbindin-1A and NF-kappa B signaling pathways. A luciferase reporter that contains NF-kappa B binding sequences in its promoter was used to characterize NF-kappa B transcription activities. RNA oligonucleotides targeting two different sequences of murine dysbindin in Neuro2A cells with knocked down dysbindin-1A exhibited markedly lower NF-kappa B transcription activities than those found in negative control cells in which p65 expression was not changed (Fig 5A and 5B).

Because NF-kappa B and dysbindin-1A are localized in the nucleus upon stimulation; and NF-kappa B activity is affected by dysbindin-1A, we wondered whether dysbindin-1A interacts with NF-kappa B directly. To address this question, we co-transfected EGFP or EGFP-p65 with Flag-dysbindin-1A into HEK293 cells or incubated GST and GST-dysbindin-1A with His-p65 *in vitro*. GST pulldown and immunoprecipitation assays showed that dysbindin-1A interacts with NF-kappa B (Fig 5C and 5D). Moreover, when endogenous dysbindin-1A immunoprecipitated with anti-dysbindin antibodies in SH-SY5Y cells, endogenous p65 was also co-immunoprecipitated (Fig 5E). To further identify the role of dysbindin-1A in nucleus, we transfected the cells with EGFP/EGFP-p65 and Flag-dysbindin-1A and performed subcellular fractionation assays. The nuclear and cytoplasmic fractions were separated and subjected to co-immunoprecipitation. The dysbindin-1A was co-immunoprecipitated with EGFP-p65 in the nuclear fraction, but not in the cytosolic fraction (Fig 5F), further suggesting an interaction between these two proteins in nucleus.

### Dysbindin-1A up-regulates MMP-9 expression in nucleus

**Fig 6:**
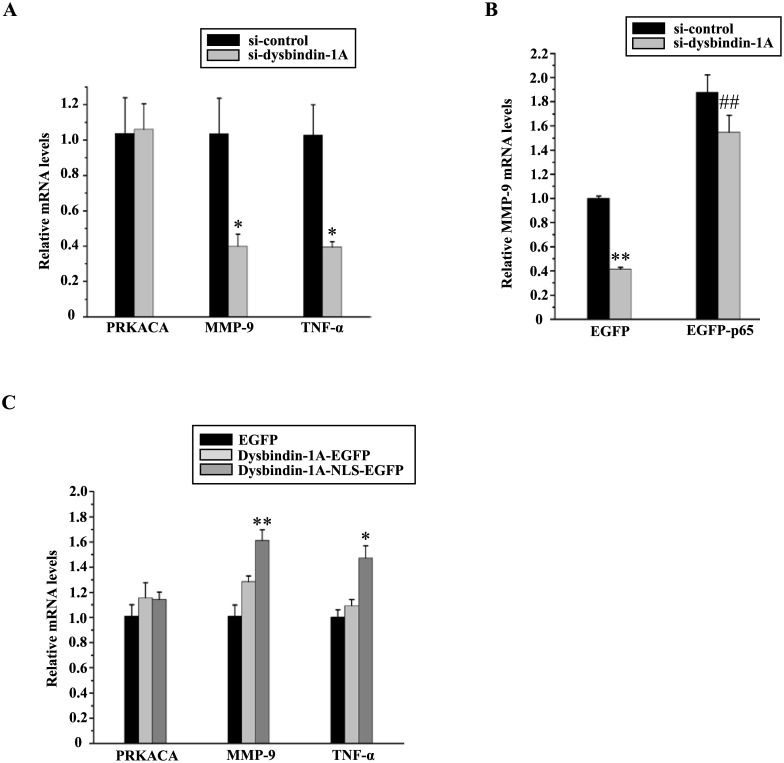
Dysbindin-1A up-regulates the MMP-9 expression. (A) NF-kappa B target gene products were examined in dysbindin-1A knockdown cells. Dysbindin-1A was silenced in N2a cells using si-dys2#, and mRNA levels of NF-kappa B downstream genes were examined using real-time PCR. The values shown represent the means ± S.E. of three independent groups. *, *P*<0.05; one-way ANOVA. (B) p65 overexpression restores gene expression resulting from dysbindin-1A deficiency. EGFP or EGFP-p65 was co-transfected with si-control or si-dys 2# into N2a cells. Forty-eight hours later, MMP-9 mRNA levels were detected using real-time PCR. The values shown represent the means ± S.E. (error bars) of three independent groups. **, *P*<0.01; EGFP/si-control vs. EGFP/si-dys; one-way ANOVA. ##, *p*<0.01; EGFP/si-dys vs. EGFP-p65/si-dys; one-way ANOVA. (C) NF-kappa B targeting gene products were examined in dysbindin-1A overexpressed cells. EGFP, dysbindin-1A-EGFP, dysbindin-1A-NLS-EGFP were transfected into N2a cells, the NF-kappa B downstream genes were examined using real-time PCR. *, *P*<0.05, **, *P*<0.01; EGFP vs. dysbindin-1A-NLS-EGFP; one-way ANOVA.

To further confirm that NF-kappa B activity is influenced by dysbindin-1A, we examined NF-kappa B-transactivated gene expression. The expression of TNF-α, a classical NF-kappa B target, was dramatically compromised when dysbindin-1A was knocked down (Fig 6A). It has been reported that PRKACA and MMP-9 are positively regulated by NF-kappa B, which plays a role in neuronal plasticity [34, 35]. Dysbindin-1A knockdown dramatically decreased MMP-9 expression, whereas PRKACA was not affected (Fig 6A). In addition, an overexpression of p65 was able to overcome the MMP-9 levels (Fig 6B).

As dysbindin-1A could bind to p65 in nucleus (Fig 5F), we wondered whether the nuclear localized dysbindin-1A could increase the NF-kappa B activity. We transfected dysbindin-1A-EGFP and dysbindin-1A-NLS-EGFP were transfected into N2a cells and analyzed the expressions of NF-kappa B downstream genes, MMP-9 and TNF-α. The dysbindin-1A with NLS significantly increased MMP-9 and TNF-α expressions (Fig 6C). These results suggest that nuclear dysbindin-1A can promote MMP-9 expression via NF-kappa B. Furthermore, we assessed whether dysbindin-1A regulates the NF-kappa B activity after deleting amino acids 2–41, which is more stable in nucleus (S1 File). Unexpectedly, the expressions of MMP-9 and TNF-α were not different in EGFP-dysbindin-1A del 2–41 and EGFP-NLS-dysbindin-1A del 2–41 groups (Fig A in S1 File). We therefore examined the interactions between EGFP-dysbindin-1A or EGFP-dysbindin-1A del 2–41 and endogenous p65 or overexpressed p65. It was shown that endogenous p65 or overexpressed p65 interacted with EGFP-dysbindin-1A, but they interacted with less EGFP-dysbindin-1A del 2–41 (Fig B in S1 File). Thus, our data suggest that a deletion of amino acids 2–41 in dysbindin-1A affects the interactions of dysbindin-1A and p65.

## Discussion

In this study, we demonstrated that dysbindin-1A is degraded in nucleus. The N-terminal amino acid sequence (residues 2–41) includes several lysines that play a role in the ubiquitination and degradation of this protein. Dysbindin-1A was found to interact with p65, a subunit of NF-kappa B, and promote its transcription factor activities. In dysbindin-1A knockdown cells, transcription of the NF-kappa B target genes MMP-9 and TNF-α was repressed.

Although the schizophrenia-related protein dysbindin-1A is primarily localized to the cytoplasm, accumulating evidence shows that it also exists in the nucleus [17–19]. As a nucleus-cytoplasm shuttling protein, dysbindin-1A is preferentially localized in the cytoplasm due to its functional NES sequences [19]. In this study, we demonstrated that the half-life of dysbindin-1A is shortened in the nucleus. Nuclear dysbindin-1A is degraded more rapidly than cytoplasmic dysbindin-1A. It has recently been reported that the dysbindin-1A interacts with TRIM32, a cytoplasmic E3 ligase that promotes substrate ubiquitination [36]. Dysbindin-1A can also be ubiquitinated when co-transfected with TRIM32 [29]. Polyubiquitination is essential for protein degradation by the proteasomal complex [37], which, although mainly present in the cytoplasm, is also present in the nucleus [30]. When dysbindin-1A and dysbindin-1A-NLS were treated with the proteasomal inhibitor MG132, the levels of these proteins were increased; in particular, a dramatic increase of nuclear dysbindin-1A was observed. The observation of increased ubiquitination of dysbindin-1A in the nucleus further supports the idea that dysbindin-1A is mainly degraded by the proteasome in the nucleus. Because the levels of many nuclear transcription factors and co-activators are highly regulated in the nucleus, nuclear dysbindin-1A might be similarly regulated [38–40]. Thus, the nucleus provides a site for dysbindin-1A degradation as well as its nuclear functions.

Lysine is a site of ubiquitin conjugation and further polyubiquitination on a substrate. Deletion of the N-terminal amino acid sequence (residues 2–41), which contains five lysines, decreased the ubiquitination of dysbindin-1A and increased its steady-state levels, suggesting that these lysines are potential ubiquitination sites and direct protein degradation. A PEST domain in the C-terminus of dysbindin-1A is involved in the proteasomal degradation of dysbindin-1A [33]. In our observations, the N-terminus, but not the C-terminus of dysbindin-1A, is ubiquitinated, suggesting a localization of the ubiquitination sites in the N-terminus. However, a deletion of the C-terminus decreases the ubiquitination of the N-terminus. Taken together, those data suggest that the C-terminus that harbors the PEST domain facilitates the ubiquitination of the N-terminus.

Dysbindin is involved in the regulation of neurodevelopment and of learning and memory. Nuclear-cytoplasmic shuttling directs the nuclear localization of dysbindin in certain states, although the conditions governing its import into the nucleus remain unknown. Several nuclear proteins that interact with dysbindin have been identified, including the transcription factor NF-YB [18]. Nuclear dysbindin-1A might function in transcriptional regulation. In this study, we found that the transcription factor NF-kappa B might be regulated by dysbindin-1A in the nucleus through direct binding to p65, a subunit of NF-kappa B. To characterize the functions of nuclear dysbindin-1A, several known targets of NF-kappa B were filtered. MMP-9, a matrix metalloproteinase that influences synaptic plasticity and learning and memory [34, 41], was decreased in dysbindin-1A depleted cells. As dysbindin-1A does not affect p65 levels, our data suggest that the interactions of dysbindin-1A and p65 may affect NF-kappa B activity but not change p65 protein level. Interestingly, a deletion of amino acids 2–41 in dysbindin-1A decreases the binding affinity of dysbindin-1A to p65, suggesting that amino acids 2–41 may be involved in dysbinin-1A interaction with p65. Dysbindin deficiency compromises spatial learning and memory function, this finding is also observed in p65 or MMP-9 knockout mice [27, 41–43]. Nuclear dysbindin-1A and NF-kappa B might therefore function in the same pathway to influence schizophrenia pathogenesis.

In summary, our study demonstrates that dysbindin-1A is degraded in nucleus and that the dysbindin nuclear-cytoplasmic shuttling property in combination with its nuclear degradation might regulate dysbindin-1A functions in regulating NF-kappa B activities in the nucleus. Thus, our data provide new insights into the role of dysbindin-1A during schizophrenia pathogenesis.

## Supporting Information

S1 File Involvement of amino acids 2–41 in dysbindin-1A in the regulation of NF-kappa B activity and fin its interaction with p65. NF-kappa B downstream gene expressions were examined in dysbindin-1A del 2–41 overexpressed cells (Fig A). Lack of amino acids 2–41 in dysbindin-1A decreased its binding to both endogenous and overexpressed p65 (Fig B).
(DOC)
