# Supplementary material for: The Schizophrenia-Related Protein Dysbindin-1A Is Degraded and Facilitates NF-Kappa B Activity in the Nucleus
Source: PLoS One. 2015 Jul 14;10(7):e0132639. doi: 10.1371/journal.pone.0132639 (PMC4501731; doi:10.1371/journal.pone.0132639)
Supplement: S1 File — NF-kappa B downstream gene expressions were examined in dysbindin-1A del 2–41 overexpressed cells (Fig A). Lack of amino acids 2–41 in dysbindin-1A decreased its binding to both endogenous and overexpressed p65 (Fig B). (DOC) [file pone.0132639.s001.doc]

**S1 File**

Involvement of amino acids 2-41 in dysbindin-1A in the regulation of NF-kappa B activity and fin its interaction with p65.


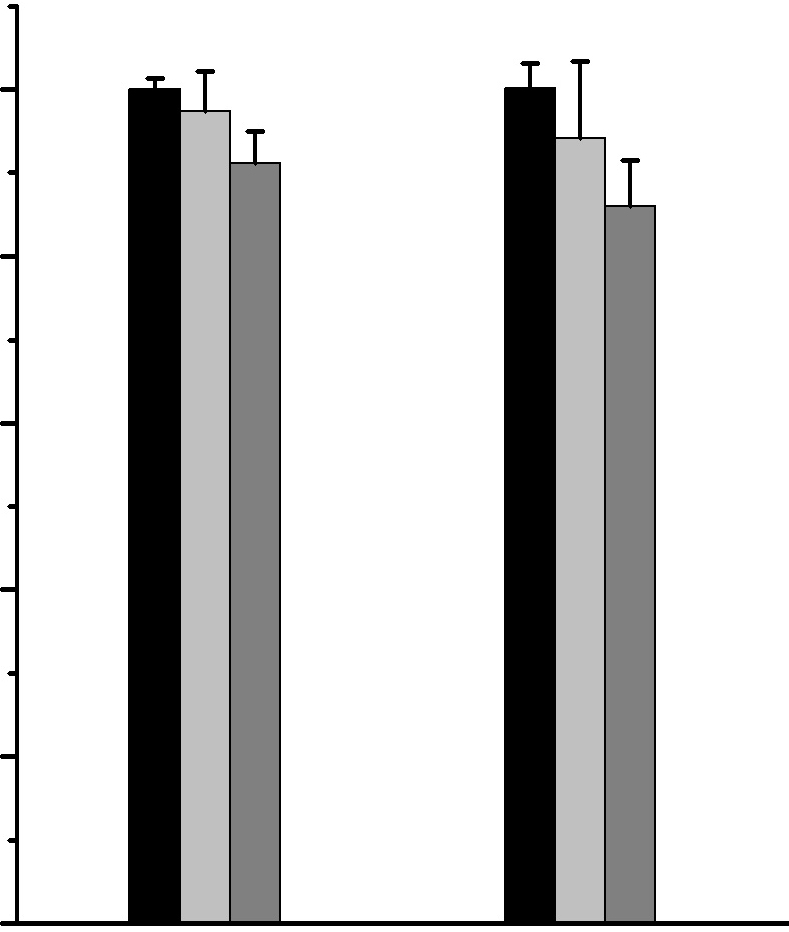


**MMP-9**

**TNF-α**

**EGFP**

**EGFP-dysbindin-1A del 2-41**

**EGFP-NLS-dysbindin-1A del 2-41**

**0**

**0.2**

**0.4**

**0.6**

**0.8**

**1.0**

**Relative mRNA levels**

Figure A. NF-kappa B downstream gene expressions were examined in dysbindin-1A del 2-41 overexpressed cells.

**+**

**-**

**-**

**+**

**-**

**+**

**-**

**+**

**-**

**-**

**+**

**+**

**EGFP**

**Dysbindin-1A-EGFP**

**EGFP-dysbindin-1A del 2-41**

**Flag-p65**


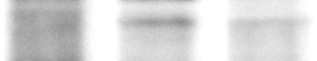


**IP: α-GFP IB: α-p65**

**Endogenous p65**


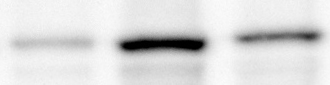


**IP: α-GFP IB: α-Flag**

**Flag-p65**


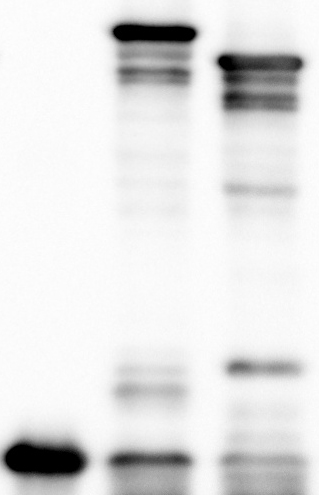


**85**

**Dysbindin-1A variants**

**IP: α-GFP**

**IB: α-GFP**

**50**

**34**

**Input IB: α-p65**

**85**

**Endogenous p65**


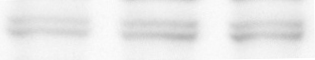


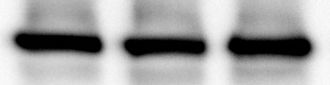


**85**

**Input IB: α-Flag**

**Flag-p65**

Figure B. Lack of amino acids 2-41 in dysbindin-1A decreased its binding to both endogenous and overexpressed p65.
